# Case Report: Whole Exome Sequencing Revealed Disease-Causing Variants in Two Genes in a Patient With Autism Spectrum Disorder, Intellectual Disability, Hyperactivity, Sleep and Gastrointestinal Disturbances

**DOI:** 10.3389/fgene.2021.625564

**Published:** 2021-02-18

**Authors:** Maria Cerminara, Giovanni Spirito, Livia Pisciotta, Margherita Squillario, Martina Servetti, Maria Teresa Divizia, Margherita Lerone, Bianca Berloco, Silvia Boeri, Lino Nobili, Diego Vozzi, Remo Sanges, Stefano Gustincich, Aldamaria Puliti

**Affiliations:** ^1^Department of Neurosciences, Rehabilitation, Ophthalmology, Genetics, Maternal and Child Health (DiNOGMI), University of Genoa, Genoa, Italy; ^2^Neuroscience Area, International School for Advanced Studies (SISSA), Trieste, Italy; ^3^Child Neuropsychiatry Unit, Azienda Socio Sanitaria Territoriale Fatebenefratelli Sacco (ASST Fbf Sacco), Milan, Italy; ^4^Medical Genetics Unit, Istituto di Ricovero e Cura a Carattere Scientifico (IRCCS) Istituto Giannina Gaslini, Genoa, Italy; ^5^Child Neuropsychiatry Unit, Istituto di Ricovero e Cura a Carattere Scientifico (IRCCS) Istituto Giannina Gaslini, Genoa, Italy; ^6^Department of Neuroscience and Brain Technologies, Istituto Italiano di Tecnologia, Genoa, Italy

**Keywords:** autism spectrum disorder, attention deficit disorder and hyperactivity, whole exome sequencing, sleep disturbance, oligenic disease, ORVAL, gut motility disorders

## Abstract

Autism Spectrum Disorder (ASD) refers to a broad range of conditions characterized by difficulties in communication, social interaction and behavior, and may be accompanied by other medical or psychiatric conditions. Patients with ASD and comorbidities are often difficult to diagnose because of the tendency to consider the multiple symptoms as the presentation of a complicated syndromic form. This view influences variant filtering which might ignore causative variants for specific clinical features shown by the patient. Here we report on a male child diagnosed with ASD, showing cognitive and motor impairments, stereotypies, hyperactivity, sleep, and gastrointestinal disturbances. The analysis of whole exome sequencing (WES) data with bioinformatic tools for oligogenic diseases helped us to identify two major previously unreported pathogenetic variants: a maternally inherited missense variant (p.R4122H) in *HUWE1*, an ubiquitin protein ligase associated to X-linked intellectual disability and ASD; and a *de novo* stop variant (p.Q259X) in *TPH2*, encoding the tryptophan hydroxylase 2 enzyme involved in serotonin synthesis and associated with susceptibility to attention deficit-hyperactivity disorder (ADHD). *TPH2*, expressed in central and peripheral nervous tissues, modulates various physiological functions, including gut motility and sleep. To the best of our knowledge, this is the first case presenting with ASD, cognitive impairment, sleep, and gastrointestinal disturbances linked to both *HUWE1* and *TPH2* genes. Our findings could contribute to the existing knowledge on clinical and genetic diagnosis of patients with ASD presentation with comorbidities.

## Introduction

Autism spectrum disorder (ASD) is a neurodevelopmental disorder characterized by etiological and clinical heterogeneity, with a frequency around 1% in the general population (Nevison et al., 2018). In the wide picture of ASD, the major clinical manifestations are communicative impairments, difficulties in social interaction, behavior abnormalities. Indeed, ASD patients frequently present with comorbidities, such as intellectual disability (ID) (Postorino et al., 2016), epilepsy (Besag, 2018), sleep or gastrointestinal disturbances (Devnani and Hegde, 2015; Bjorklund et al., 2020; Brooks et al., 2020).

The genetic basis of ASD is deeply heterogeneous implicating different genes, in turn involved in different pathways and biological processes, as those regulating synaptic plasticity, chromatin remodeling, gene transcription, and protein degradation (Bourgeron, 2015). Single nucleotide changes or larger genomic alterations, as copy number variations (CNVs), can be found in ASD cases (Sanders et al., 2015), and recent studies proved that the use of Whole Exome Sequencing (WES) together with CNVs analysis can identify a pathogenetic variant in about 30% of patients (Munnich et al., 2019).

We here report on a male child with ASD and a complex phenotype, who was analyzed by WES with a bioinformatic tool for investigating variants in oligogenic diseases. The results suggest a hypothetical scenario in which two genes (*HUWE1* and *TPH2*) plus 4 possible modifiers could play a role in the patient's phenotype.

## Methods

### Clinical Assessment

The clinical assessment of the patient comprised a neurological-behavior examination and evaluation of his sleep and gastrointestinal disturbances as detailed in Supplementary Material.

### Whole Exome Sequencing

Genomic DNA extraction and subsequent WES and data analysis for affected individual and his parents were carried out by Italian Institute of Technologies (IIT) in Genoa, Italy. Briefly, the exomes were captured using the xGen® Exome Research Panel v1.0 – IDT KIT. Sequences were enriched using the Illumina Nextera Flex For Enrichment KIT. Sequencing was performed on an Illumina NovaSeq 6000 platform (Illumina Inc., CA, USA). The library preparation and its sequencing were performed simultaneously for the parents and the proband using barcode adapters. Alignment of raw paired-end reads to the reference genome (version hg19) was performed with bwa (version 0.7.17) (Li and Durbin, 2010). Duplicated reads were marked with picard (version 2.18.20). Variant discovery was then performed with the GATK4 utility HaplotypeCaller, using the appropriate file containing the coordinates of the sequences targeted by the exome sequencing. Finally all variants were annotated using annovar (databases updated to 27/05/2019) (Wang et al., 2010). The resulting file was used for a manual evaluation of the variants. The sequencing provided a 60x medium coverage.

### Variant Prioritization

For the interpretation of variants pathogenicity, we integrated several tools. To unveil the contribution of multiple genes to the patient's phenotype, we used a new platform for the prediction and exploration of candidate disease-causing oligogenic variant combinations (ORVAL, Oligogenic Resource for Variant AnaLysis) following authors recommendations (Renaux et al., 2019). Briefly, we prepared two files: (i) an input file with a list of selected candidate variants and (ii) one file with genes related or possibly related to ASD to be used as “gene panel” file for variant filtering.

The list of candidate variants was obtained by considering the following filtering steps: variants with a frequency below 3% in ExAC/GnomAD v2.11/1000g2015; only exonic, splicing, non-synonymous and stopgain variants with a good coverage were considered; we discarded those variants predicted to be benign or tolerated in PolyPhen and SIFT and retained those with CADD score ≥ 20; we also discarded those variants found in more than 1% of in-house controls (i.e., not diagnosed with ASD or any neuropsychiatric disease). Filtering was also based on annotation in public databases as Mouse Genome Database (MGD), OMIM, PubMed, ClinVar. Following this procedure we obtained a list of 71 candidate variants, including both *de novo* and inherited, to be used as input in ORVAL.

The “gene panel” file consisted of genes selected based on three criteria: those related to ASD and present in the SFARI (Simons Foundation Autism Research Initiative) database (August 2020 release); genes with high brain expression, defined as those genes with average log2 RPKM >4.5 in the BrainSpan database (http://www.brainspan.org) (the top 18%) (Miller et al., 2014; Addis et al., 2018) and genes of the KEGG pathways (https://www.genome.jp/kegg) selected for their possible relevance with ASD.

From the output file, a list of possible 23 variants, we kept only those genes/variants characterized by pathogenicity confidence score higher than 99%. These selected genes underwent through a manual revision considering literature and available databases. Briefly, we analyzed all the selected output variants for deleterious prediction through Varsome (Kopanos et al., 2019), we considered the intolerance to loss-of-function variants (pLI) and the deviation of the observed number of missense variants from the expected number (Mis Z-score), as computed by the Exome Aggregation Consortium (ExAC) (http://exac.broadinstitute.org/) (genes with pLI scores of 0.9 or higher are extremely intolerant to heterozygous LoF variation, and thus haploinsufficient; Z-scores of 3.09 or higher indicate intolerance to missense variation) (Lek et al., 2016), we also considered haploinsufficiency on the base of an HIPred_score above 0.5 (Shihab et al., 2017). Finally, a list of 6 variants in 6 genes was obtained.

Selected variants in the candidate genes were all validated by co-segregation analysis using polymerase chain reaction (PCR), and bi-directional Sanger sequencing using the ABI 3730 automated sequencer (Applied Biosystems, Forster City, CA, USA).

### Network Analysis of Candidate Genes

To unveil enrichment of annotations of identified genes and known ASD genes, we used GeneCodis4 tool (Tabas-Madrid et al., 2012), as already described (Vaccari et al., 2016). Briefly, a list enclosing all 6 candidate genes, obtained from the ORVAL analysis and that passed our manual inspection, together with all genes present in the SFARI database (August 2020 release) were used as input in GeneCodis4 (total genes = 934). Among the databases of biological knowledge available in GeneCodis4, we focused on the GO Biological Process (BP) domain. In the analysis, the hypergeometric test was applied followed by the false discovery rate correction (FDR) with a cut-off of 5% to determine which annotations were significantly enriched. All genes of the top gene ontology terms, including candidate and SFARI genes, were then projected onto the STRING network (v11) (Szklarczyk et al., 2019). Edges within the STRING network were thresholded at 0.4, according to the authors' recommendation. A graphical representation of the GeneCodis4 and STRING results was obtained by using Cytoscape tool (Shannon et al., 2003).

## Case Presentation

**Figure 1:**
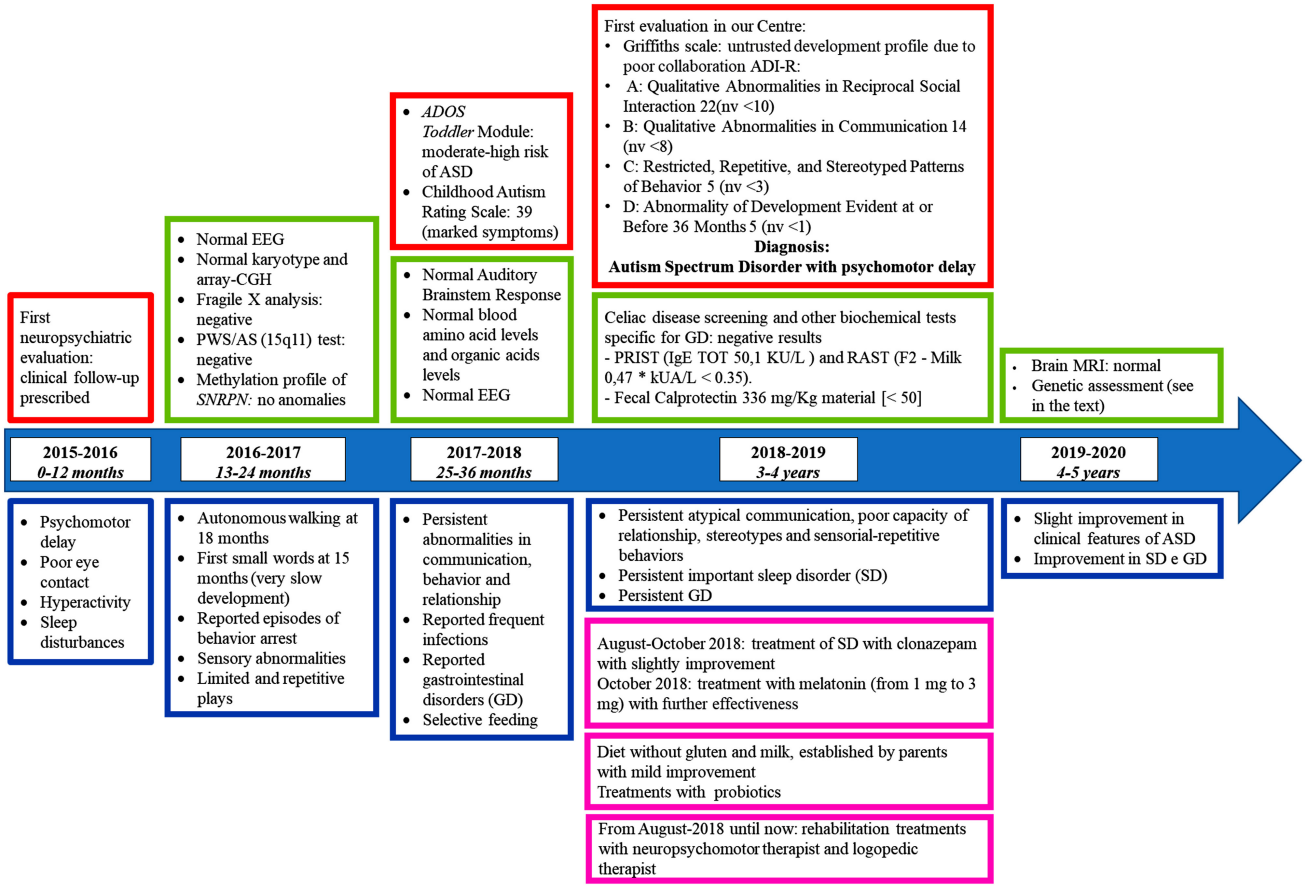
Timeline with patient's relevant clinical data. In this figure main signs and symptoms (blue) shown by the patient, data from his clinical evaluations (red), results of laboratory tests (pink), and treatments (green) are reported. PWS/AS, Prader-Willi Syndrome/Angelman Syndrome; GD, gastro-intestinal disorders; SD, sleep disorders; ADOS, Autism Diagnostic Observation Schedule; ADI-R, Autism Diagnostic Interview-Revised; ASD, Autism Spectrum Disorder; PRIST, Paper Radio Immuno Sorbent Test; RAST, Radio Allergo Sorbent Test; MRI, Magnetic Resonance Imaging; nv, normal value.

We report on a 5-years old male patient born to non-consanguineous Italian parents. The child came to our attention at the age of 3 years for third level investigations, when he had already performed several clinical assessments and tests at the territorial structures (see the timeline, Figure 1). After a thorough medical history and after having viewed and analyzed the clinical documentation, he was diagnosed with ASD even by the help of Autism Diagnostic Observation Schedule (ADOS) and the Autism Diagnostic Interview-Revised (ADI-R). He had developmental delay, absent language, motor impairment, auto and hetero-direct aggressivity, hyperactivity, attentional lability, stereotypes.

In the past, metabolic defects and the main syndromes in differential diagnosis were excluded, Fragile X Syndrome and Prader-Willi/Angelman Syndromes (see timeline, Figure 1).

More in details, the clinical assessment of the patient through specific tests showed a low adaptive level for chronological age, prevailing on communication and socialization domains, a deficit on ability of inhibit and self-control inhibition, metacognition, working memory, and ability of organization/planning, problems in the area of withdrawal and isolation, anxiety and depression, somatic complaints, and attentional disturbances, marked impairments on language understanding and production.

By using specific tests (Sleep-CGI-S, Sleep-CGI-I, SDSC) the patient was diagnosed with a moderate sleep disorder. Sleep-CGI-S showed a moderate sleep disorder with marked bedtime resistance, moderate sleep onset delay, moderate difficult to fall asleep, multiple night wakings and family functioning moderately affected. Instead Sleep-CGI-I reported minimal improving in ability to fall asleep, a major improving in bedtime resistance, sleep onset delay, night wakings, and family functioning. At SDSC the results confirmed the mentioned sleep disorders. In particular, the patient has been treated with clonazepam for 2 months and then, until now, with melatonin, that slightly improved his condition (see timeline, Figure 1).

Using the Criteria of Rome IV, functional constipation (3 criteria) and functional aerophagia (4 criteria) were diagnosed.

Results obtained from neurological-behavior tests and of sleep evaluation are reported in Supplementary Table 1.

## Results

### Identification of Candidate Genetic Variants

Given the complexity of the patient's phenotype, we suspected a possible genetic basis involving multiple genes. Accordingly, for the identification of candidate variants, we used a bioinformatic tool for the study of oligogenic diseases.

**Figure 2:**
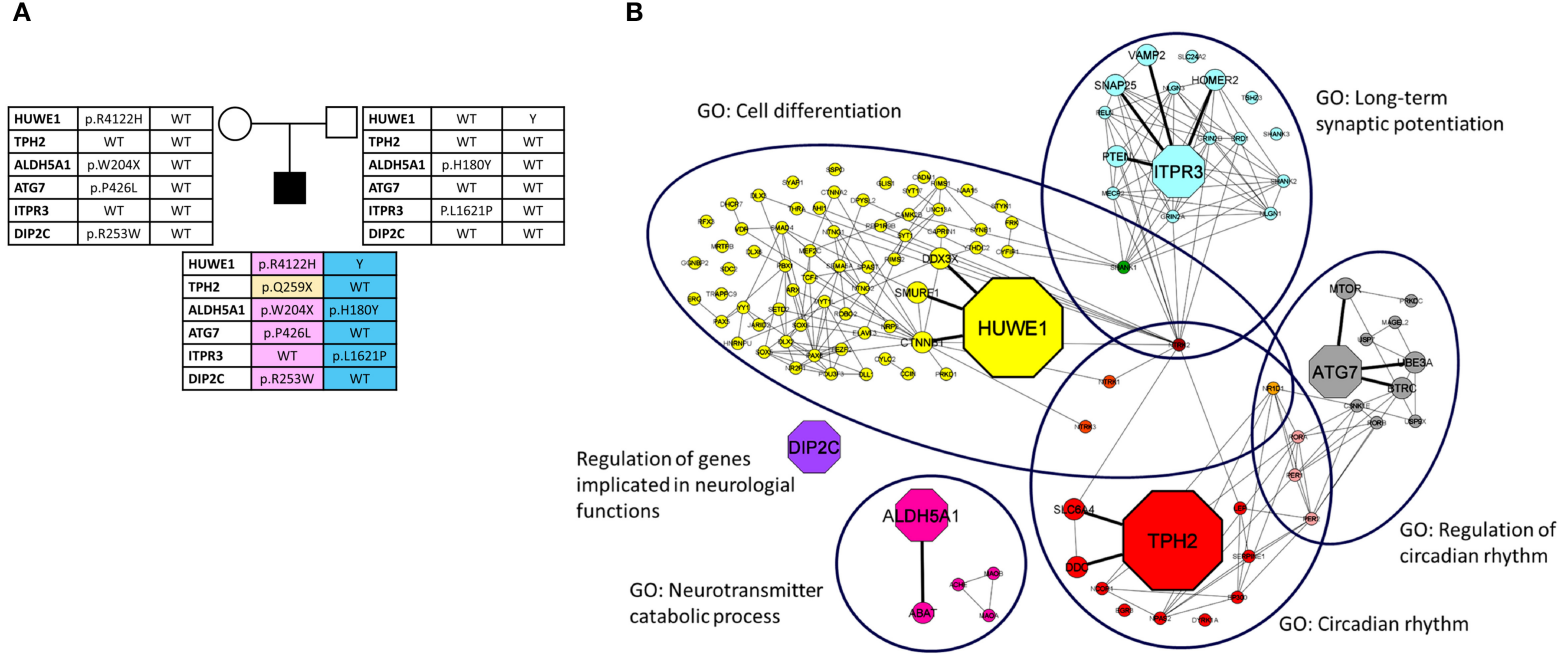
**(A)** Family's pedigree. Implicated genes and their variants, indicated as amino acid changes, are shown. **(B)** Network analysis. The network depicts the protein-protein interactions (gray lines) revealed by STRING analysis (visualized by Cytoscape tool) on the basis of the enrichment obtained with GeneCodis4 tool. The Figure shows the enriched BP terms clustering the six candidate genes (octagon shape) and the SFARI genes (circle shape). Three candidate genes are also in the SFARI database (*HUWE1, ALDH5A1, DIP2C*). The network has a clear distinction into 6 clusters representing the top six GO-BPs (large ovals).

After the bioinformatic analysis of WES data an unreported maternally inherited missense variant affecting the E3 ubiquitin ligase (HECT) domain of *HUWE1* (NM_031407:exon79: c.12365G>A:p.R4122H), and a *de novo* unreported stop variant in *TPH2* (NM_173353:exon6: c.775C>T:p.Q259X) (see Figure 2A, Table 1) were identified.

**Table 1:** Overview of the variants found in the patient.

**Gene**	**Position**	**Fnc**	**RefSeq ID**	**Variation**	**GnomAD**	**1000G**	**SIFT**	**Poly-phen2**	**CADD**	**dbSNP ID**	**Var**	**pLI**	**Z score**	**Hapl-insuf**
HUWE1^*^	X: 53563401	M	NM_031407	c.12365G>A:p.R4122H	NA	NA	D	D	27.7	NA	LP	1	8.87	0.831
*TPH2*	12: 72366465	Sg	NM_173353	c.775C>T:p.Q259X	NA	NA	–	–	40	NA	VUS	0	1	0.653
*ALDH5A1#*	6: 24505099	Sg	NM_170740	c.612G>A:p.W204X	0.00004	NA	–	–	41	rs118203982	P	0	0.73	0.505
*ALDH5A1#*	6:24503590	M	NM_170740	c.538C>T: p.H180Y	0.3105	0.3147	T	B	7.5	rs2760118	B	0	0.73	0.505
*ITPR3*	6: 33651870	M	NM_002224	c.4862T>C:p.L1621P	0.00001	NA	D	D	29.3	rs1217162248	VUS	0	4.55	0.603
*DIP2C°*	10: 461811	M	NM_014974	c.757C>T:p.R253W	NA	NA	D	D	34	NA	VUS	1	4.67	0.563
*ATG7*	3: 11389502	M	NM_006395	c.1277C>T:p.P426L	0.00120	0.0004	D	D	34	rs143545741	VUS	0	1.37	0.652

*Fnc, Function; M, Missense; Sg, Stopgain; NA, not available; D, deleterious; T, tolerated; CADD score, amino acid substitution is predicted damaging if the score is >15; Var, Varsome; VUS, Variant of Uncertain Significance; B, Benign; LP, Likely Pathogenic; P, Pathogenic; GnomAD is referred to GnomAD Exome ALL*.

* *, SFARI gene, syndromic “S”; #, SFARI gene, score 1; °, SFARI gene, score 2*.

*HUWE1* encodes an ubiquitin protein ligase involved in various cellular processes, including synaptogenesis, associated to the Turner type X-linked syndromic cognitive disability (OMIM:309590) and ASD (Moortgat et al., 2018). The *TPH2* gene encodes the tryptophan hydroxylase 2 enzyme involved in serotonin synthesis in central and peripheral nervous tissues. Serotonin modulates various physiological functions, including regulation of gut motility and sleep (Jones et al., 2020). *TPH2* variants are associated to susceptibility to attention deficit-hyperactivity disorder (OMIM:613003) and unipolar depression (OMIM:608516).

The analysis unveiled the presence of additional variants in the following genes, *ALDH5A1, ATG7, ITPR3*, and *DIP2C*.

The *ALDH5A1* variant is a rare, maternally inherited stop variant (NM_170740:exon4: c.612G>A:p.Trp204X). *ALDH5A1* encodes the succinic semialdehyde dehydrogenase, implicated in the neurotransmitter GABA catabolism (Chambliss et al., 1995). Homozygous or compound heterozygous mutations in *ALDH5A1* cause the succinic semialdehyde dehydrogenase deficiency (OMIM:271980), a rare complex neurologic disorder mainly characterized by mental retardation and sleep disturbances. By further investigating WES data, we unveiled an additional, paternally inherited, variant in *ALDH5A1* (NM_170740:exon3:c.538C>T:p.His180Tyr). This last variant, present in the general population with a frequency of 0.3154, was considered a hypomorphic variant, leading to a reduction of *ALDH5A1* enzymatic activity of about 20% (Blasi et al., 2002).

The *ITPR3* variant is a paternally inherited, rare missense variant (NM_002224:exon36: c.4862T>C:p.Leu1621Pro). *ITPR3* encodes for the inositol 1,4,5-trisphosphate receptor that transduces many hormonal signals regulating Ca(2+)-dependent processes. To note, the mouse carrying the *tf* spontaneous mutation of the *Itpr3* gene (BTBR T+ Itpr3tf /J strain) has been used as a model of ASD (McFarlane et al., 2008).

The patient showed a maternally inherited unreported missense variant of *DIP2C* (NM_014974:exon7: c.757C>T:p.Arg253Trp). *DIP2C* encodes a member of the disco-interacting protein homolog 2 family, known to regulate several genes and pathways important in neurological functions (Oo et al., 2020). To note, in ASD cases *de novo* loss of function variants were found in *DIP2C*, which is reported in SFARI database as candidate ASD gene (Yuen et al., 2017).

The maternally inherited *ATG7* variant is a rare missense variant (NM_006395:exon12: c.1277C>T:pPro426Leu). *ATG7* encodes an ubiquitin-activating enzyme E1-like protein that mediates membrane fusion in autophagy (Tanida et al., 2001). Autophagy deficiency induced by conditional Atg7 deletion leads to a similar autistic-like behavioral abnormalities in mice (Hui and Tanaka, 2019). *ATG7* plays a role in modulation of dendritic arborization, and synapses elimination during development, mechanisms relevant to the etiology of neurodevelopmental disorders as ASD (Kim et al., 2017).

We investigated the role of the 6 candidate genes in already known ASD-associated biological processes and their possible connection among each other and with other ASD-associated genes reported in SFARI database.

The results indicated that each of the analyzed genes, except *DIP2C*, takes part to at least one process in which other SFARI genes are involved, namely “long term synaptic potentiation,” “neurotransmitter catabolic process,” “circadian rhythm,” “regulation of circadian rhythm,” “cell differentiation” (Figure 2B, Table 2).

**Table 2:** Biological processes associated with genes implicated in the present case and genes reported in the SFARI database.

**Annotation**	**Term**	**Term genes found**	**Input size**	**Term genes**	**Genes universe**	**Hyp pval**	**Hyp pval_adj**	**Genes**
GO:0060291	Long-term synaptic potentiation	17	934	45	18,883	0.0000	0.0000	SLC24A2, SHANK2, NLGN1, VAMP2, SNAP25, PTEN, RELN, NTRK2, MECP2, **ITPR3**, GRIN2B, GRIN2A, SHANK3, DRD1, TSHZ3, NLGN3, SHANK1
GO:0060999	Positive regulation of dendritic spine development	10	934	23	18,883	0.0000	0.0000	ZMYND8, SHANK2, NLGN1, **DLG5**, CDKL5, ITSN1, MTOR, FMR1, SHANK3, SHANK1
GO:0007623	Circadian rhythm	17	934	91	18,883	0.0000	0.0001	NCOR1, NR1D1, PER2, SLC6A4, RORA, PER1, SERPINE1, NTRK3, NTRK2, NTRK1, NPAS2, LEP, **TPH2**, EP300, EGR3, DYRK1A, DDC
GO:0042752	Regulation of circadian rhythm	14	934	66	18,883	0.0000	0.0002	**ATG7**, NR1D1, BTRC, PER2, USP9X, USP7, UBE3A, RORB, RORA, PRKDC, PER1, MTOR, MAGEL2, CSNK1E
GO:0042135	Neurotransmitter catabolic process	5	934	10	18,883	0.0001	0.0021	**ALDH5A1**, ACHE, MAOB, MAOA, ABAT
GO:0030154	Cell differentiation	70	934	1,019	18,883	0.0036	0.0423	CADM1, SYNE1, CYFIP1, SSPO, MYT1L, UNC13A, RIMS1, NTNG1, **HUWE1**, RIMS2, NR1D1, CCIN, SEMA5A, NRP2, CAMK2B, YY1, VDR, THRA, NR2F1, TCF4, SYT1, SPAST, SOX5, SDC2, ROBO2, RFX3, PRKD1, POU3F3, PBX1, PAX6, PAX5, NTRK3, NTRK2, NTRK1, MEF2C, SMAD4, CAPRIN1, JARID2, HNRNPU, FRK, ARX, GLIS1, SYAP1, ERG, PPP1R9B, NTNG2, ELAVL3, TRAPPC9, NAA15, GGNBP2, DPYSL2, YTHDC2, DLX6, DLX3, DLX2, DHCR7, MRTFB, SMURF1, DDX3X, SOX6, STYK1, FEZF2, CYLC2, AHI1, CTNNB1, CTNNA2, SYT17, SHANK1, SETD2, DLL1

*Term genes found, number of annotated genes in the reference list; Input size, total number of genes in the input list, including the genes implicated in the present case and the SFARI list genes; Term genes, number of annotated genes in the reference list; Genes universe, total number of human reference genes; Hyp pval, hypergeometric test pValue; Hyp pval_adj, corrected hypergeometric pValue using FDR procedure*.

## Discussion and Conclusion

ASD is a complex and heterogeneous condition characterized by impaired communication and social interaction, repetitive behaviors, and restricted interests and associated with a range of comorbid conditions. Intellectual disabilities can affect around 50% of ASD cases (Postorino et al., 2016), ADHD is present in about 11% of children and even higher in adult ASD patients (Lugo et al., 2020), and sleep and gastrointestinal disturbances have elevated prevalence in ASD cases compared to controls (Devnani and Hegde, 2015; Bjorklund et al., 2020). The genetic base of ASD is also heterogeneous, involving at least 102 risk genes (Satterstrom et al., 2020). ASD-associated variants can be rare or more common in the general population. Finally, compensatory mechanisms can tune the major or the minor effect of the variations determining the phenotype present in each subject (Bourgeron, 2015).

In our case, the patient presented ASD associated with intellectual disability, ADHD, sleep, and gastrointestinal disorder. By a deep bioinformatic analysis of WES data we identified *de novo* and inherited variants in six different genes that globally could explain the complex phenotype shown by this patient.

The *HUWE1* p.R4122H variant lies in the HECT domain, known to present an overrepresentation of deleterious variants in patients with X-linked ID (Moortgat et al., 2018). *In vitro* experiments demonstrated that mutations in the HECT domain are likely to impair the gene function (Giles and Grill, 2020). Indeed, certain *HUWE1* variants are present in patients with autism in addition to ID, suggesting *HUWE1* variants could be risk factors for ASD (Giles and Grill, 2020). In the present case, the *HUWE1* variant could account for ID and contribute to ASD together with the other gene candidates. *TPH2* is involved in the synthesis of serotonin, a neurotransmitter with a role in the control of mood, anxiety, sleep-wake regulation and, peripherally, in the modulation of gastrointestinal motility. It has been reported that behavioral symptoms can be associated with gastrointestinal abnormalities in ASD patients, and, in particular, that ASD patients with sleep problems are more likely to have gastrointestinal abnormalities (Maenner et al., 2012; Bjorklund et al., 2020). In this line, we suggest that the *TPH2 de novo* stop variant could account for the hyperactivity, attentional lability, sleep and gastrointestinal disturbances present in this patient.

For function and pattern of inheritance, *HUWE1* and *TPH2* could play a major role in the patient's phenotype.

Variants were also identified in *ALDH5A1*, whose absence of activity has been associated to a recessive severe phenotype. In the present case, we could speculate that the stop and the hypomorphic variants leave a quantity of expressed enzyme lower than that expressed by a heterozygous carrier, in this way contributing to the patient's phenotype (Oikonomou et al., 2019; Jones et al., 2020).

Three additional deleterious variants were found, inherited from either patient's healthy mother (*ATG7, DIP2C*) or his healthy father (*ITPR3*) suggesting that taken individually, in heterozygosis, these variants do not lead to altered neurodevelopment. Since gene enrichment analysis highlighted they take part to functions notably impaired in ASD, we are unable to exclude they play a role in the patient's phenotype. Rather, they could contribute to the patient's complex phenotype as modifier genes.

To note, increasing findings support an oligogenic model of inheritance for ASD, and combination of inherited and/or *de novo* variants were predicted to range from 2 to 10 (Pickles et al., 1995). However, genetic variants, their connections and how they can act in every single patient, are aspects still largely to be elucidated.

In conclusion, by using an oligogenic approach to the analysis of WES data, we identified a *de novo* stop variant in *TPH2* and one deleterious missense variant in *HUWE1* that could act in a permissive genetic background characterized by deleterious variants in additional four genes. All variants collectively could have a role in the biological processes involved in the pathogenesis of ASD thus contributing to the different features of the patient's phenotype. As this hypothesis is based on the study of a single patient and on variants predicted to be deleterious by bioinformatic analyses, future functional studies are needed to elucidate specific roles of identified genes and variants in the patient's complex phenotype.

## Data Availability Statement

Clinical and genetic variant details have been deposited in the Decipher database. Accession number: Patient 421248.

## Ethics Statement

The studies involving human participants were reviewed and approved by the Ethics Committee of the Italian Regione Liguria (P.R. 399REG2017). Written informed consent to participate in this study was provided by the participants' legal guardian/next of kin. Written informed consent was obtained from the individual(s), and minor(s) legal guardian/next of kin, for the publication of data included in this article.

## Author Contributions

GS, DV, RS, and SG were responsible for whole exome sequencing. MC, MSq, MSe, and AP were responsible for the variant prioritization, oligogenic and network analyses. LP, BB, SB, and LN were in charge of the clinical diagnosis. MD, ML, and AP were in charge of genetic counseling, family recruitment, and ethical procedures. LP and AP wrote the manuscript. All authors gave advises when doing the gene analysis, reviewed the manuscript, and agreed to be accountable for the content of the work.

## Conflict of Interest

The authors declare that the research was conducted in the absence of any commercial or financial relationships that could be construed as a potential conflict of interest.
