# Supplementary material for: Case Report: Whole Exome Sequencing Revealed Disease-Causing Variants in Two Genes in a Patient With Autism Spectrum Disorder, Intellectual Disability, Hyperactivity, Sleep and Gastrointestinal Disturbances
Source: Front Genet. 2021 Feb 18;12:625564. doi: 10.3389/fgene.2021.625564 (PMC7930735; doi:10.3389/fgene.2021.625564)
Supplement: Supplementary file 1 [file Table_1.DOCX]

**SUPPLEMENTARY MATERIAL**

**Clinical assessment**

The clinical assessment of the patient comprised a neurological-behavior examination and evaluation of his sleep and gastrointestinal disturbances.

Psychomotor evaluations were assessed administering Griffiths Mental Development Scales (GMDS), third edition, a comprehensive, child-friendly developmental measure for continuous use from birth to 6 years (Griffiths, 1984). Adaptive skills were tested by administering to the parents the Vineland Adaptive Behavior Scales-Second Edition (VABS-II), an interview that assesses communication, daily living and social skills. The Vineland-II Adaptive Behavior Composite (VABC) score, with three subscales and their nine subdomains (Communication: receptive, expressive and written subdomain; Daily living: personal, domestic and community subdomain; Socialization: interpersonal relationships, play-leisure time and coping skills subdomain) were reported in Supplementary table 1 (Sparrow and Cicchetti, 1985; de Bildt et al., 2005).

Executive functions were assessed using the BRIEF-P Behavior Rating Inventory of Executive Function–Preschool Version (Ezpeleta et al., 2015). BRIEF is an assessment of executive function behaviors at home and at school for children and adolescents. The 63-item questionnaire has separate forms for parents and teachers.

Behavior disturbances were evaluated using the CBCL Child Behavior Checklist (Achenbach and Dumenci, 2001).

New Reynell Developmental Language Scales (NRDLS) was used to assess child’s language ability and to evaluate the effectiveness of interventions (Letts et al., 2013).

To evaluate the degree of sleep disorders, Pediatric Sleep and Autism Clinical Global Impressions Scales and Scale-Improvement (Sleep CGI-S and Sleep CGI-I), and the Sleep Disturbance Scale for Children (SDSC) were administered (Bruni et al., 1996; Busner and Targum, 2007). The Sleep CGI-S is a scale that healthcare professionals use to rate the patient’s severity of sleep disorder at the time of assessment, relative to the clinician’s past experience with patients who have the same diagnosis. The Sleep CGI-I requires the clinician to assess how much the patient’s disorder has improved or worsened relative to a baseline state at the beginning of an intervention.

The SDSC is a 27-item inventory rated on a 6 point Likert-type scale: disorders of initiating and maintaining sleep, sleep breathing disorders, disorders of arousal, sleep-wake transition disorders, disorders of excessive somnolence, and sleep hyperhidrosis.

To evaluate the degree of gastro-intestinal disorders Roma Criteria for gastrointestinal disorders were administered (Drossman and Hasler, 2016). The "Criteria di Roma", produced and recently overhauled by an international working group (Rome IV criteria) provide guidelines for the diagnosis of Functional gastrointestinal disorders. The diagnostic criteria of Roma IV for functional gastrointestinal disorders are: functional disorders of the esophagus, functional disorders of the stomach and duodenum, functional intestinal disorders, functional abdominal pain syndrome, functional disorders of the gallbladder and sphincter of Oddi, functional diseases of the anus and rectum, functional disorders in infants and young children (0-4 years), functional disorders in older children and adolescents (5-18 years), functional disorders associated with abdominal pain, constipation and faecal incontinence.

Achenbach, T.M., and Dumenci, L. (2001). Advances in empirically based assessment: revised cross-informant syndromes and new DSM-oriented scales for the CBCL, YSR, and TRF: comment on Lengua, Sadowksi, Friedrich, and Fischer (2001). *J Consult Clin Psychol* 69(4)**,** 699-702.

Bruni, O., Ottaviano, S., Guidetti, V., Romoli, M., Innocenzi, M., Cortesi, F., et al. (1996). The Sleep Disturbance Scale for Children (SDSC). Construction and validation of an instrument to evaluate sleep disturbances in childhood and adolescence. *J Sleep Res* 5(4)**,** 251-261. doi: 10.1111/j.1365-2869.1996.00251.x.

Busner, J., and Targum, S.D. (2007). The clinical global impressions scale: applying a research tool in clinical practice. *Psychiatry (Edgmont)* 4(7)**,** 28-37.

de Bildt, A., Sytema, S., Kraijer, D., Sparrow, S., and Minderaa, R. (2005). Adaptive functioning and behaviour problems in relation to level of education in children and adolescents with intellectual disability. *J Intellect Disabil Res* 49(Pt 9)**,** 672-681. doi: 10.1111/j.1365-2788.2005.00711.x.

Drossman, D.A., and Hasler, W.L. (2016). Rome IV-Functional GI Disorders: Disorders of Gut-Brain Interaction. *Gastroenterology* 150(6)**,** 1257-1261. doi: 10.1053/j.gastro.2016.03.035.

Ezpeleta, L., Granero, R., Penelo, E., de la Osa, N., and Domenech, J.M. (2015). Behavior Rating Inventory of Executive Functioning-Preschool (BRIEF-P) Applied to Teachers: Psychometric Properties and Usefulness for Disruptive Disorders in 3-Year-Old Preschoolers. *J Atten Disord* 19(6)**,** 476-488. doi: 10.1177/1087054712466439.

Griffiths, R. (1984). *The Abilities of Young Children: A Comprehensive System of Mental Measurement for the First Eight Years of Life. Revised ed. .*

Letts, C., Edwards, S., Sinka, I., Schaefer, B., and Gibbons, W. (2013). Socio-economic status and language acquisition: children's performance on the new Reynell Developmental Language Scales. *Int J Lang Commun Disord* 48(2)**,** 131-143. doi: 10.1111/1460-6984.12004.

Sparrow, S.S., and Cicchetti, D.V. (1985). Diagnostic uses of the Vineland Adaptive Behavior Scales. *J Pediatr Psychol* 10(2)**,** 215-225. doi: 10.1093/jpepsy/10.2.215.

**Supplementary Table 1.** Results obtained from neurological-behavior tests and of sleep evaluation.

| TEST | RESULTS | RANGE | INTERPRETATION |
| --- | --- | --- | --- |
| VABC score | 45 | 0-100 | low adaptive level |
| Communication domain | 43 | 0-100 | low adaptive level |
| Daily Living Skill domain | 56 | 0-100 | low adaptive level |
| Socialization domain | 50 | 0-100 | low adaptive level |
| Motor skills domain | 63 | 0-100 | low adaptive level |
| BRIEF-P overall composite score (T-score) | 77 | T < 50 normal,  T > 65 abnormal | abnormal |
| Inhibition (T-score) | 77 | T < 50 normal,  T > 65 abnormal | abnormal |
| Shift (T-score) | 62 | T < 50 normal,  T > 65 abnormal | probably abnormal |
| Regulation of emotions (T-score) | 66 | T < 50 normal,  T > 65 abnormal | abnormal |
| Working memory (T-score) | 73 | T < 50 normal,  T > 65 abnormal | abnormal |
| Planning / organization (T-score) | 74 | T < 50 normal,  T > 65 abnormal | abnormal |
| Inhibitory self-control index (T-score) | 75 | T < 50 normal,  T > 65 abnormal | abnormal |
| Flexibility Index (T-score) | 66 | T < 50 normal,  T > 65 abnormal | abnormal |
| Emerging metacognition index (T-score) | 75 | T < 50 normal,  T > 65 abnormal | abnormal |
| CBCL overall score (T-score) | 74 | T < 65 normal,  T > 70 abnormal | abnormal |
| Emotional reactions (T-score) | 70 | T < 65 normal,  T > 70 abnormal | abnormal |
| Anxiety and depression (T-score) | 70 | T < 65 normal,  T > 70 abnormal | abnormal |
| Somatic complaints (T-score) | 65 | T < 65 normal,  T > 70 abnormal | normal |
| Withdrawal / isolation (T-score) | 85 | T < 65 normal,  T > 70 abnormal | abnormal |
| Sleep disturbances (T-score) | 70 | T < 65 normal,  T > 70 abnormal | abnormal |
| Attention disorders (T-score) | 70 | T < 65 normal,  T > 70 abnormal | abnormal |
| Aggressive behavior (T-score) | 64 | T < 65 normal,  T > 70 abnormal | normal |
| NRDLS understanding | 69 1st percentile (age equivalent <2 years) | 0-100th percentiles | abnormal |
| NRDLS production | 69 1st percentile (age equivalent <2 years) | 0-100th percentiles | abnormal |
| Sleep Disturbance Scale for Children  total score (T score) | 72 | T < 65 normal,  T > 70 abnormal | abnormal |
| Disorders of initiating and maintaining sleep  (T score) | 93 | T < 65 normal,  T > 70 abnormal | abnormal |
| Sleep breathing disorders (T score) | 45 | T < 65 normal,  T > 70 abnormal | normal |
| Disorders of arousal (T score) | 70 | T < 65 normal,  T > 70 abnormal | abnormal |
| Sleep-wake transition disorders (T score) | 54 | T < 65 normal,  T > 70 abnormal | normal |
| Disorders of excessive somnolence (T score) | 93 | T < 65 normal,  T > 70 abnormal | abnormal |
| Sleep hyperhidrosis (T score) | 45 | T < 65 normal,  T > 70 abnormal | normal |

Note: VABC = Vineland Adaptive Behavior Composite score; BRIEF-P = Behavior Rating Inventory of Executive Function–Preschool Version; CBCL = Child Behavior Checklist; NRDLS = New Reynell Developmental Language Scale
